# Protected Areas in Tropical Africa: Assessing Threats and Conservation Activities

**DOI:** 10.1371/journal.pone.0114154

**Published:** 2014-12-03

**Authors:** Sandra Tranquilli, Michael Abedi-Lartey, Katharine Abernethy, Fidèle Amsini, Augustus Asamoah, Cletus Balangtaa, Stephen Blake, Estelle Bouanga, Thomas Breuer, Terry M. Brncic, Geneviève Campbell, Rebecca Chancellor, Colin A. Chapman, Tim R. B. Davenport, Andrew Dunn, Jef Dupain, Atanga Ekobo, Manasseh Eno-Nku, Gilles Etoga, Takeshi Furuichi, Sylvain Gatti, Andrea Ghiurghi, Chie Hashimoto, John A. Hart, Josephine Head, Martin Hega, Ilka Herbinger, Thurston C. Hicks, Lars H. Holbech, Bas Huijbregts, Hjalmar S. Kühl, Inaoyom Imong, Stephane Le-Duc Yeno, Joshua Linder, Phil Marshall, Peter Minasoma Lero, David Morgan, Leonard Mubalama, Paul K. N'Goran, Aaron Nicholas, Stuart Nixon, Emmanuelle Normand, Leonidas Nziguyimpa, Zacharie Nzooh-Dongmo, Richard Ofori-Amanfo, Babafemi G. Ogunjemite, Charles-Albert Petre, Hugo J. Rainey, Sebastien Regnaut, Orume Robinson, Aaron Rundus, Crickette M. Sanz, David Tiku Okon, Angelique Todd, Ymke Warren, Volker Sommer

**Affiliations:** 1 Department of Biological Anthropology, University College London, London, United Kingdom; 2 Department of Migration and Immuno-Ecology, Max Planck Institute for Ornithology, Radolfzell, Germany; 3 Department of Biology, University of Konstanz, Konstanz, Germany; 4 African Forest Ecology Group, School of Natural Sciences, University of Stirling, Stirling, United Kingdom; 5 IRET, Libreville, Gabon; 6 Frankfurt Zoological Society, Maiko National Park, Tshopo, Democratic Republic of Congo; 7 Environmental Sustainability Project, United Nations Development Programme/Ghana Cocoa Board, Adabraka, Accra, Ghana; 8 Wildlife Division of Forestry Commission of Ghana, Ankasa Conservation Area, Elubo, Takoradi, Ghana; 9 Wildlife Conservation Society, New York, United States of America; 10 Whitney Harris World Ecology Center, University of Missouri – Saint Louis, Saint Louis, Missouri, United States of America; 11 Ministère de la Forêt, de l'Environnement et de la Protection des Ressources Naturelles, Libreville, Gabon; 12 Wildlife Conservation Society, Congo Program, Brazzaville, Republic of Congo; 13 Tacugama Chimpanzee Sanctuary, Freetown, Sierra Leone; 14 Independent Researcher, Montreal, Canada; 15 Departments of Anthropology & Sociology, and Psychology, West Chester University, West Chester, Pennsylvania, United States of America; 16 Department of Anthropology and McGill School of Environment, McGill University, Montréal, Québec, Canada; 17 Wildlife Conservation Society, Tanzania Program, Zanzibar, Tanzania; 18 Wildlife Conservation Society, Calabar, Nigeria; 19 African Wildlife Foundation, Nairobi, Kenya; 20 World Wide Fund for Nature, Limbe, Cameroon; 21 World Wide Fund for Nature, Mount Cameroon NP, Limbe, Cameroon; 22 World Wide Fund for Nature CARPO, Jengi Tridom, Yaundé, Cameroon; 23 Primate Research Institute, Kyoto University, Kyoto, Japan; 24 Support for Conservation of Bonobos, Luo Reserve, Democratic Republic of Congo; 25 West African Primate Conservation Action, Accra, Ghana; 26 Independent Researcher, Rome, Italy; 27 Lukuru Wildlife Research Foundation, Kinshasa, Democratic Republic of Congo; 28 Department of Primatology, Max Planck Institute for Evolutionary Anthropology, Leipzig, Germany; 29 Wildlife Conservation Society, Monts de Cristal, Gabon; 30 World Wide Fund for Nature, Berlin, Germany; 31 Institute for Biodiversity and Ecosystem Dynamics, The University of Amsterdam, Amsterdam, The Netherlands; 32 Department of Animal Biology and Conservation Science, University of Ghana, Legon, Accra, Ghana; 33 World Wide Fund for Nature, Central Africa Regional Programme Office, Yaoundé, Cameroon; 34 German Centre for Integrative Biodiversity Research, Leipzig, Germany; 35 World Wide Fund for Nature, Gamba, Libreville, Gabon; 36 Department of Sociology and Anthropology, James Madison University, Harrisonburg, Virginia, United States of America; 37 North Eastern Parks Programme, Windhoek, Namibia; 38 Directorate of Wildlife Service, Ministry of Interior and Wildlife Conservation, Juba, Republic of South Sudan; 39 Lester E. Fisher Center for Great Ape Research, Lincoln Park Zoo, Chicago, Illinois, United States of America; 40 World Wide Fund for Nature, Itombwe Conservation Programme, Bukavu, South Kivu Province, Eastern Democratic Republic of Congo; 41 Centre Suisse de Recherches Scientifiques en Côte d’Ivoire, Abidjan, Côte d’Ivoire; 42 Wildlife Conservation Society, Ruaha-Katavi Landscape, Tanzania; 43 Zoological Society of London, London, United Kingdom; 44 Wild Chimpanzee Foundation, Abidjan, Cote d’Ivoire; 45 Institut National pour l'Environnement et la Conservation de la Nature, Bururi, Burundi; 46 Bia Conservation Area, Sefwi Wiawso, Ghana; 47 Department Ecotourism and Wildlife Management, Federal University of Technology, Akure, Nigeria; 48 Laboratory of Tropical and Subtropical Forestry, Unit of Forest and Nature Management, Gembloux Agro-Bio Tech, University of Liège, Gembloux, Belgium; 49 Education and Nature, Conservation Biology Unit, Royal Belgian Institute of Natural Sciences, Brussels, Belgium; 50 International Union for Conservation of Nature, Protected Areas Program West and Central Africa, Ouagadougou, Burkina Faso; 51 Ministry of Forestry and Wildlife, Korup National Park, Ndian, Cameroon; 52 Department of Psychology, West Chester University, West Chester, Pennsylvania, United States of America; 53 Department of Anthropology, Washington University, Saint Louis, Missouri, United States of America; 54 World Wide Fund for Nature, Korup National Park, Limbe, Cameroon; 55 World Wide Fund for Nature, Bangui, Central African Republic; 56 Wildlife Conservation Society, Limbe, Cameroon; Institute of Agronomy, University of Lisbon, Portugal

## Abstract

Numerous protected areas (PAs) have been created in Africa to safeguard wildlife and other natural resources. However, significant threats from anthropogenic activities and decline of wildlife populations persist, while conservation efforts in most PAs are still minimal. We assessed the impact level of the most common threats to wildlife within PAs in tropical Africa and the relationship of conservation activities with threat impact level. We collated data on 98 PAs with tropical forest cover from 15 countries across West, Central and East Africa. For this, we assembled information about local threats as well as conservation activities from published and unpublished literature, and questionnaires sent to long-term field workers. We constructed general linear models to test the significance of specific conservation activities in relation to the threat impact level. Subsistence and commercial hunting were identified as the most common direct threats to wildlife and found to be most prevalent in West and Central Africa. Agriculture and logging represented the most common indirect threats, and were most prevalent in West Africa. We found that the long-term presence of conservation activities (such as law enforcement, research and tourism) was associated with lower threat impact levels. Our results highlight deficiencies in the management effectiveness of several PAs across tropical Africa, and conclude that PA management should invest more into conservation activities with long-term duration.

## Introduction

Tropical rainforests harbour a particularly rich and unique biodiversity [1]. Though representing only 7% of land surface, they support more than 60% of all known species [2]. However, their existence is compromised by many interrelated anthropogenic threats that have intensified over recent decades [3], [4]. Increased human population growth and economic expansion have fostered the rapid expansion of two of the main threats to wildlife, habitat destruction and unsustainable hunting. These disturbances have caused several declines in wildlife populations and have contributed to the degradation of many tropical forests [5], [6], [7]. Over the past 20–30 years, threats to African tropical forests in particular have attracted national and international attention. This has led to the creation of numerous protected areas (PAs), which are intended to conserve both fauna and flora, whilst benefitting neighbouring human communities [8], [9], [10].

Nevertheless, human populations throughout Africa have increased the amount of pressure being exerted on PAs. Thus, despite their legal protected status, PAs face significant threats. Of particular concern are overexploitation of natural resources, habitat loss, fragmentation and isolation (e.g., [6],[11]–[14]). These factors impact severely on key species and especially taxa with large body sizes, slow reproductive rates, and little behavioural adaptability [15], [16]. Moreover, many PAs in tropical Africa are “paper-parks” where conservation efforts are minimal or non-existent [17]. Thus, many wildlife populations continue to decline [18] and local extinctions have become increasingly common within PAs (e.g., colobus monkeys: [19]; great apes: [20], [21]; ungulates: [22]).

The persistence of wildlife in a PA depends largely on the magnitude of anthropogenic pressures and the success of conservation efforts to combat such threats [17]. For instance, the mere continuous presence of conservation non-governmental organizations (NGOs), law enforcement, tourism or research in PAs has a positive effect on the persistence of large mammals within PAs, such as apes [23]. Conversely, inadequate law enforcement, including insufficient training, guard numbers, equipment, patrols and funding enables poaching and other illegal activities [6], [24], [25].

Many PAs are not adequately funded by the national governments, because they are not considered to be economically viable investments [26]. Therefore, they often depend on additional support from both international and national NGOs working in partnerships with national and regional authorities. Nevertheless, the total funding allocated to a PA is often insufficient to ensure effective protection [26], [27], [28].

The management performance, threat impact level and wildlife status inside African PAs have been increasingly assessed over the last decade [6], [23], [29]. These evaluations are particularly important to bridge the gap between policy makers, funding bodies and conservation practitioners [30]. However, threats to PAs in tropical areas are notoriously difficult to assess due to poor or non-existent data and typically limited to case studies (e.g., [31], [32]) or individual countries [33], [34]. In addition, the significance of conservation activities in relation to the impact level of the different threats to PAs has rarely been evaluated on a broad scale in tropical Africa.

To address this deficit, we collected information on threats to wildlife and conservation activities in 98 PAs in tropical forests throughout Africa with significant wildlife populations. In this study we evaluate the impact levels of 12 different threats both at a continental and regional scale. Moreover, we use an evidence-based approach to assess the relative significance of a range of conservation activities (including law enforcement, tourism and research activities) that are aimed at the protection of PAs. Based on our findings, we provide recommendations to enhance effective conservation management.

## Methods

### Ethical statement

The research was reviewed and approved by the Ethical Review Process of the University College London (UCL) before data collection began.

The ethics committee allowed the involvement of human participants in the project and their participation in written, face-to-face and phone interviews, and a written consent declaring their willingness to participate in this study and to be a co-author of the study. All the participants provided via e-mail written informed consent to participate in the study and to be a co-author.

### Data collection

**Figure 1:**
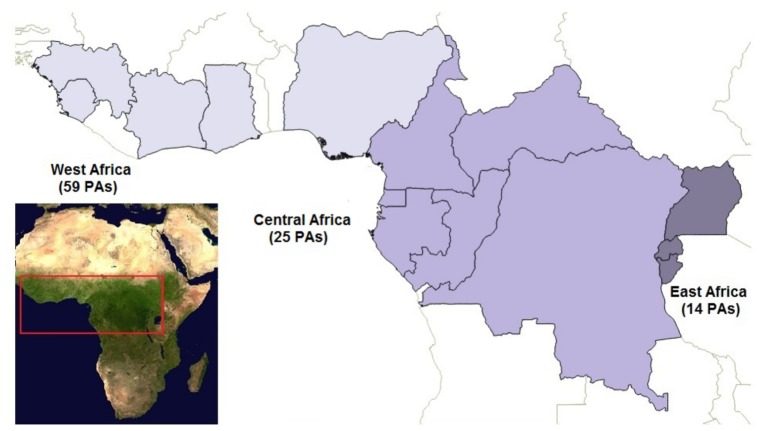
Regional distribution of the protected areas (PAs) in tropical Africa considered in the analyses. The regions are coloured in different grey scale colours. Light grey represents West Africa, including 54 protected areas; medium grey represents Central Africa, including 31 protected areas; dark grey represents East Africa, including 14 protected areas. On the left-side bottom corner a MODIS NDVI image of Africa, with a red quadrant highlighting the tropical area considered in the study.

We collected information about local threats to wildlife and conservation activities of 98 PAs from 14 countries with tropical forest cover (Fig. 1, Table S1), across West, Central and East Africa. These areas harbour significant wildlife populations, including endangered and charismatic large and small mammals (such as apes, elephants, leopards, monkeys, and pangolins) and birds (such as vultures and eagles) [35]. Size of PAs ranged from 9.3 to 32, 967 km^2^ covering a total area of 182, 797 km^2^. The PAs included in the analyses focussed on the conservation of wildlife and ecosystem services.

Data on local threats to wildlife in PAs were collected from published literature and a list of the 12 common known threats was generated, as cited in the literature of the past 20 years as the most critical to wildlife within PAs (Table 1, Text S1). These include (i) direct threats, with short-term and immediate effects on wildlife populations (i.e. removal and killing of individual animals) such as subsistence and commercial hunting, and (ii) indirect threats, with long-term effects that drive wildlife population declines, such as illegal agriculture, collection of fuel wood, fire, mining, logging and human settlement density, infrastructure, armed conflicts and disease.

**Table 1:** Common threats to wildlife in protected areas in tropical Africa and their definition.

Direct Threats	Definition
Subsistence hunting	Illegal killing of wildlife by locals inside the protected area to supplement scarce diet.
Commercial hunting	Illegal killing and/or capture of wildlife by locals or outsiders inside the protected area for commercial purposes (i.e. to sell the meat in large markets of villages or cities for consumption as delicacy or for pet trade).
**Indirect Threats**	**Definition**
Agriculture	Illegal conversion of forest land inside the protected area for agriculture purpose.
Disease	Presence of disease outbreak in wildlife populations inside the protected area originated from humans.
Fire	Illegal use of fire to create cattle pasture or to enable agriculture inside the protected area.
Collection of fuel wood	Illegal extraction of forest wood from the protected area for use as firewood and/or charcoal.
Infrastructure	Road construction and use by vehicles inside the protected area.
Logging	Illegal cutting, on-site processing and harvest of timber from the protected area.
Mining	Illegal extraction of mineral resources from the protected area.
Human settlements inside	Presence of villages inside the protected area.
Human settlements around	Presence of villages within a buffer of 50 km from the border of the area.
Armed conflicts	Country armed conflict or war in action

In addition, questionnaires about the generated list of common threats were completed by long-term field workers and augmented by face-to-face or phone interviews (Text S1, Table S2). Interviewees were asked to score the relative importance of individual threats to wildlife populations for the years of the last decade when they worked at their respective sites. Threats were assigned to the following impact levels: level 0 (absent impact), level 1 (low impact, threat present with minimal impact on wildlife populations), level 2 (moderate impact, threat present and affecting wildlife populations with impact not critical to their survival), level 3 (high impact, threat present with impact critically affecting the survival of wildlife populations). Moreover, interviewees were invited to describe additional “site-specific” or “country-specific” threats.

Data on conservation activities in PAs for each year were collected from published and unpublished literature, and gaps in information for specific areas were filled by the expert knowledge of conservation scientists and practitioners through questionnaires or phone interviews (Text S1, Tables S3, S4). These data aimed to cover the last 20 years for 105 PAs and included primary activities (direct actions to reduce threats; i.e. law enforcement) and secondary activities (indirect supportive actions, i.e. tourism and research). We selected these conservation activities for the analyses on the basis of a recent study that demonstrated their important role in reducing species extinction risk in tropical Africa [23].

### Statistical analyses

Correlations between the impact levels of two threats at a time were investigated on a continent-wide scale, employing Spearman's correlations test and a post-hoc Bonferroni correction to reduce the error of multiple testing. To visualize in detail the relationships of similarities and dissimilarities in threats we developed dendrograms, as a graphical representation of the matrix of Euclidian distances between groups of threats.

We calculated the percentage of PAs having a particular threat at different impact levels, to reveal any differences at a continental and regional level. In addition, we analyzed the proportion of PAs, both at a continental and at regional scale in tropical Africa, to investigate the temporal trend of conservation activities presence over the last two decades.

We used General Linear Models (GLMs) [36] with binomial error structure and logit link function to evaluate the relative importance of conservation activities variables on ‘PA conservation status'. We used the term ‘PA conservation status’ to refer to the level of impact of several threats to its wildlife and thus to its ecological viability, having a binary status ‘not threatened/threatened with more than 30% of threats at level 2 and 3′. We analyzed all possible GLM subsets for the three categories of test variables, i.e., law enforcement, tourism, and research. In addition, we included the size of the PA as control variable, assuming that the threat impact levels vary according to the area dimension (see Table 2 for the description of the predictor variables). In addition, we ran a correlation between all conservation activities of Table 2 (Table S5) and eliminated the most redundant, and then we used both forward and backward stepwise regression analysis to identify the strongest predictors of threat impact level among all the three conservation activity categories [37]. These latter analyses were based on a dataset where information on each conservation activity was available for each PA for the period considered, and encompassed 76 PAs. All variables were *z*-transformed before model fitting. For both GLMs and stepwise regression models, we used the Akaike's Information Criterion (AIC) model selection [38], [39] to identify the predictor variables that best explained the data. The Akaike Information Criterion Weight (AICw) was calculated for each model to obtain the one that best explains the relative explanatory value of the different predictors influencing the response variable [40]. All analyses were conducted using R software (version 2.11.1; [41]). The GLMs and stepwise regression models were carried out using the function “lmer” from R package “lme4” [42], and the function “step” from R package “MASS” [43], respectively.

**Table 2:** Predictor variables considered in the GLM analyses.

Predictor category	Predictor variable	Abb.^a^	Definition
PA characteristic	PA size^**^	S	Area in square kilometers
Law enforcement	Guards^*^	G	Proportion of years with guards present
	Number of guards^*^	NG	Average number of guards employed
	Guards monthly patrol^*^	MP	Proportion of years when guards went on monthly patrols.
Research	Research site^*^	R	Proportion of years with researcher program present
	Research station^*^	RS	Average number of months with operative research station per year
Tourism	Tourism site^*^	T	Proportion of years with tourism present
	Tourist station^*^	TS	Average number of months with operative tourism station per year
	Number of tourists^*^	NT	Average number of tourist visitors per year

(^a^) Abbreviation used in models (see Tab 4, 5, 6)

(^*^) Test variables included information during the five years prior to the year when PA threat impact level was scored, as an approximation of temporal change of these variables, between 1992 and 2011 (source: literature, questionnaires).

(^**^) Control variables (source: World Database on Protected Areas).

## Results

### Relationships between threats

A total of 66 correlations between the threat impact levels were performed for the overall sample of 98 PAs (Table 3). Impact levels of subsistence and commercial hunting were positively correlated, likewise subsistence hunting and agriculture. Moreover, there was a positive correlation between the combined impacts of agriculture and fuel wood collection on one hand, and agriculture and human settlement on the other. Additional positive correlations were found between the level of impact of fuel wood collection and the use of fire (Table 3).

**Table 3:** Symmetric matrix with Spearman's correlation between all threat impact levels recorded in 98 protected areas.

	coh	suh	agr	fuw	inf	hsa	hsi	war	dis	fir	min	log
**coh**	1.00											
**suh**	**0.55**	1.00										
**agr**	0.07	**0.52**	1.00									
**fuw**	0.22	0.36	**0.60**	1.00								
**inf**	0.20	0.25	0.40	0.32	1.00							
**hsa**	0.15	0.40	**0.48**	0.36	0.39	1.00						
**hsi**	0.03	0.31	**0.55**	0.39	0.25	0.41	1.00					
**war**	−0.08	0.08	0.28	0.12	0.22	0.04	0.31	1.00				
**dis**	0.06	0.10	−0.05	0.06	−0.09	0.20	0.10	0.11	1.00			
**fir**	0.17	0.34	0.30	**0.50**	0.14	0.37	0.38	−0.05	0.11	1.00		
**min**	0.37	0.25	−0.01	0.15	0.31	0.31	0.04	−0.05	0.22	0.32	1.00	
**log**	0.30	0.34	0.27	0.38	0.40	0.13	0.11	0.23	−0.07	0.11	0.21	1.00

In bold are highlighted significant correlations (*p*<0.0001) following post hoc test Bonferroni correction (*p* = 0.05/78). Abbreviations: coh, commercial hunting; suh, subsistence hunting; agr, agriculture; fuw, fuel wood; inf, infrastructure; has, human settlement around; his, human settlement inside; war, war; dis, disease; fir, fire; min, mining; log, logging.

The dendrogram showed the associations between three main clades (Fig. S2). The geometry indicated that groupings within each clade were more similar to each other than to those within any other clade. War and disease were shown to be dissimilar to the rest of the other threats (clade I); agriculture and collection of wood activities were close to each other and belonging to the same clade with human settlements within and around PAs (clade II). Close relationship was shown for both the two hunting activities and infrastructure and logging, in contrast to fire and mining (clade III).

### Threat impact level at a continental and regional scale

**Figure 2:**
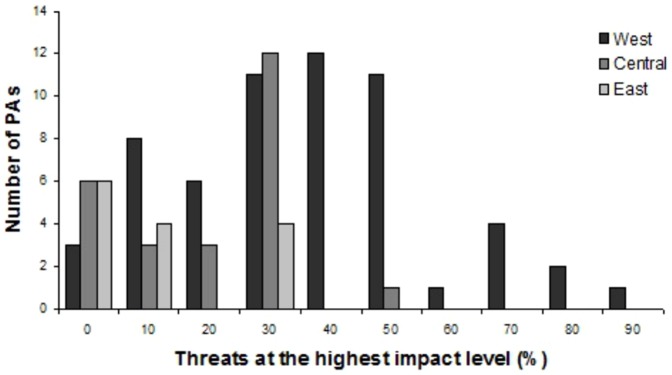
Number of protected areas with percentage of threats at the highest impact level per region.

Wildlife populations within 83 out of the 98 PAs (85%) were rated as highly threatened, with at least one threat at level 3. Moreover, 32 PAs experienced more than 40% of all the 12 listed threats at level 3. Of these, 31 sites were located in West Africa (Fig. 2).

**Figure 3:**
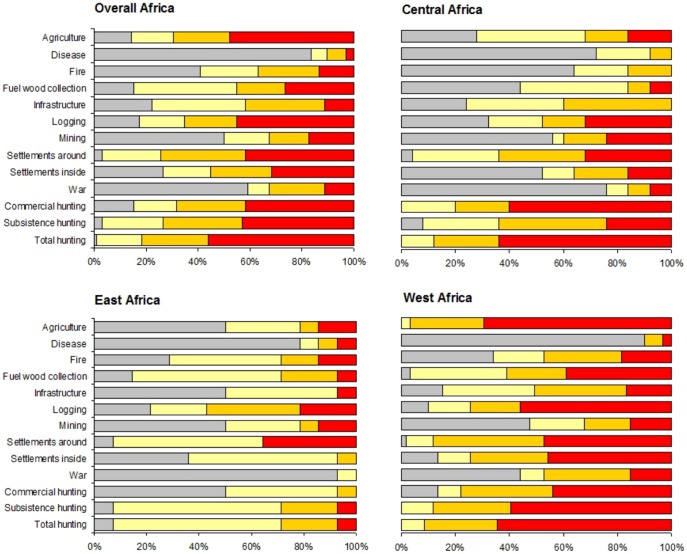
Threats impact levels to 98 tropical African protected areas at a continental and regional scale. Clockwise from top: Africa (a), Central Africa (b), East Africa (c) and West Africa (d).

Among the threats at impact level 3 across tropical Africa, hunting was the most common for 56% of all PAs (Fig. 3a). However, little difference was found between subsistence and commercial hunting (42% and 41% of sites, respectively). Agriculture and logging were the most common indirect threats with rank at level 3 in 48% and 45% of all the sites, respectively. Human settlements within and bordering PAs had also high impact on wildlife for 31% and 41% of the areas (Fig. 3a).

Concerning the regional distribution of threats, West Africa harboured the most threatened PAs suffering threats ranked at level 3 (95% of sites; n  = 59 PAs) (Fig. 3d). Specifically, the direct threats most frequently rated with the highest impact were hunting (with subsistence hunting being slightly more prevalent than commercial hunting). As for indirect threats, agriculture and logging were the most common high impact threats.

Central Africa (n = 25 PAs) showed a similar scenario with respect to hunting. However, commercial hunting was found to be more prevalent, with the highest impact level than subsistence hunting. Human settlements around the PAs and logging had the highest percentage of high impact level within the indirect human threats (32% of the areas; Fig. 3b).

In East Africa (n = 14 PAs), subsistence hunting was scored at the highest impact level in contrast to commercial hunting, although at a small percentage (7%; Fig 3c). Human density around the PAs was found to be the most prevalent threat with impact level 3 in relation to other activities (36% of the sites; Fig. 3c).

When interviewees described additional threats, cattle grazing was the most common threat added and this was present in both West Africa (12 sites, with 66% of them at level 3, occurring in Nigeria, Sierra Leone and Côte d'Ivoire) and East Africa (one site in Rwanda at level 3). Presence of refugees, and human-wildlife conflicts were perceived as threats principally in Uganda (Fig. S1).

### Conservation activities and influence on threat levels

**Figure 4:**
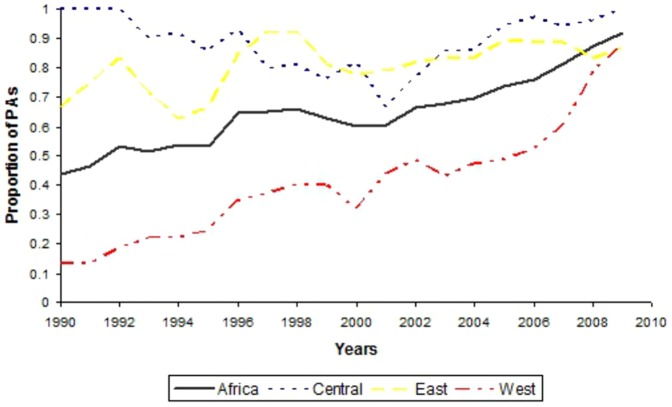
Proportion of protected areas with conservation activities between 1990 and 1999 across different African regions. The number of protected areas with available information on presence and absence of any conservation activity (research, tourism and law enforcement guards) over the considered period were in total 105.

The last two decades have seen a considerable increase of conservation activities across tropical forest Africa (Fig. 4). During the 1990s, West Africa had the lowest presence of conservation activities; however, PAs with conservation activities reached similar proportion in all regions towards the end of the first decade of the new millennium (Fig. 4).

Concerning law enforcement activities, the proportion of years with the presence of guards was significant in all the models where it was included. The model that best explained the relationship of law enforcement on the threat impact level in a PA included the proportion of years with guards being present as a single test variable (*guards ± SE* = *−0.962*±*0.368, p =  0.009, n = 90, AIC_w_* = *0.306*; Table 4).

**Table 4:** Influence of law enforcement activities and PA size on threat levels in 90 PAs.

		Coefficients					Parameters			
Model variables		Intercept	S	G	NG	MP	AIC	AICw	Rank	k
S	*Estimate*	1.731	−0.622				80.321	0.011	7	2
	*(SE)*	(0.310)	(0.291)							
	*P value*	**2.54e^−8^**	**0.032**							
S+G	*Estimate*	2.062	−0.576	−0.962			73.672	0.306	1	3
	*(SE)*	(0.408)	(0.329)	(0.368)						
	*P value*	**4.35e^−7^**	0.079	**0.009**						
S+NG	*Estimate*	1.7311	−0.627		0.0213		82.315	0.004	9	3
	*(SE)*	(0.310)	(0.299)		(0.271)					
	*P value*	**2.54e^−8^**	**0.036**		0.937					
S+MP	*Estimate*	1.803	−0.575			−0.449	79.916	0.014	6	3
	*(SE)*	(0.329)	(0.296)			(0.292)				
	*P value*	**4.59e^−8^**	0.052			0.124				
S+NG+MP	*Estimate*	1.826	−0.635		0.275	−0.576	81.127	0.007	8	4
	*(SE)*	(0.335)	(0.305)		(0.332)	(0.323)				
	*P value*	**5.31e^−8^**	**0.037**		0.407	0.075				
S+G+NG	*Estimate*	2.137	−0.703	−1.153	0.433		73.752	0.294	2	4
	*(SE)*	(0.429)	(0.348)	(0.399)	(0.352)					
	*P value*	**6.56e^−7^**	**0.043**	**0.003**	0.218					
S+G+MP	*Estimate*	2.109	−0.624	−1.353		0.478	74.389	0.214	3	4
	*(SE)*	(0.420)	(0.337)	(0.503)		(0.418)				
	*P value*	**5.35e^−7^**	0.064	**0.007**		0.253				
S+G+NG+MP	*Estimate*	2.160	−0.719	−1.409	0.363	0.347	75.112	0.149	4	5
	*(SE)*	(0.436)	(0.352)	0.511	(0.353)	(0.432)				
	*P value*	**7.29e^−7^**	**0.040**	**0.006**	0.3033	0.422				

In bold are highlighted significant values (p *<0.05*). See abbreviations in Tab 2. AIC, Akaike's Information Criterion; AICw, Akaike Information Criterion weight; Rank, model rank from the smallest to the largest AIC value; k, number of variables including the intercept.

As for the relationship with research activities, the average number of months with an operative research station was found to be the most significant (research station *± SE  = −0.573*±*0.266, p =  0.031, n = 92*, *AIC_w_  = 0.416;* Table 5). Moreover, significant association was evident for the presence of research alone as a single test variable (Table 5).

**Table 5:** Influence of research activities and PA size on threat level in 92 PAs.

		Coefficients				Parameters			
Models		Intercept	S	R	RS	AIC	AICw	Rank	k
S	*Estimate*	1.741	−0.572			81.780	0.008	4	1
	*(SE)*	(0.307)	(0.291)						
	*P value*	**1.38e^−8^**	**0.049**						
S+R	*Estimate*	1.878	−0.399	−0.652		79.353	0.104	2	3
	*(SE)*	(0.343)	(0.315)	(0.326)					
	*P value*	**4.49e^−8^**	0.205	**0.045**					
S+RS	*Estimate*	1.838	−0.385		−0.573	79.209	0.416	1	3
	*(SE)*	(0.328)	(0.314)		(0.266)				
	*P value*	**2.19e^−8^**	0.220		**0.031**				
S+R+RS	*Estimate*	1.885	−0.342	−0.417	−0.348	80.191	0.168	3	4
	*(SE)*	(0.344)	(0.322)	(0.407)	(0.330)				
	*P value*	**4.06e^−8^**	0.288	0.306	0.292				

In bold are highlighted significant values (p *<0.05*). See abbreviations in Tab 2. AIC, Akaike's Information Criterion; AICw, Akaike Information Criterion weight; Rank, model rank from the smallest to the largest AIC value; k, number of variables including the intercept.

Concerning tourism activities, the model which included the average number of months with the duration of an active tourist station best explained the relative importance of tourism *(tourist station ± SE* = *− 0.508*±*0.264, p =  0.054, n = 83, AIC_w_* = *0.257;* Table 6). In addition to the influence of the various conservation activities, the larger size of the PA was associated with lower threat impact level. This was shown in particular in models were it was present as a single predictor variable but also in models with various test variables (Tables 4, 5, and 6).

**Table 6:** Influence of tourism activities and PA size on threat level in 83 PAs.

		Coefficients					Parameters			
Models		Intercept	S	T	TS	NT	AIC	AICw	Rank	k
S	*Estimate*	1.740	−0.690				73.639	0.119	4	1
	*(SE)*	(0.327)	(0.298)							
	*P value*	**9.8e^−8^**	**0.021**							
S+T	*Estimate*	1.840	−0.643	−0.487			72.588	0.200	2	3
	*(SE)*	(0.353)	(0.308)	(0.276)						
	*P value*	**1.93e^−7^**	**0.037**	0.078						
S+TS	*Estimate*	1.836	−0.624		−0.508		72.093	0.257	1	3
	*(SE)*	(0.351)	(0.304)		(0.264)					
	*P value*	**1.72e^−7^**	**0.040**		**0.054**					
S+NT	*Estimate*	1.742	−0.685			−0.0478	75.604	0.044	8	3
	*(SE)*	(0.327)	(0.299)			(0.252)				
	*P value*	**9.98e^−8^**	**0.022**			0.850				
S+TS+NT	*Estimate*	1.844	−0.624		−0.631	0.254	73.414	0.133	3	4
	*(SE)*	(0.353)	(0.305)		(0.304)	(0.349)				
	*P value*	**1.71e^−7^**	**0.040**		**0.038**	0.467				
S+T+TS	*Estimate*	1.843	−0.620	−0.196	−0.356		73.933	0.102	5	4
	*(SE)*	(0.353)	(0.307)	(0.478)	(0.452)					
	*P value*	**1.83e^−7^**	**0.044**	0.680	0.431					
S+T+NT	*Estimate*	1.849	−0.651	−0.566		0.179	74.203	0.089	6	4
	*(SE)*	(0.355)	(0.309)	(0.303)		(0.312)				
	*P value*	**1.99e^−7^**	**0.035**	0.062		0.566				
S+T+TS+NT	*Estimate*	1.854	−0.620	−0.242	−0.454	0.272	75.166	0.055	7	5
	*(SE)*	(0.356)	(0.309)	(0.471)	(0.456)	(0.356)				
	*P value*	**1.85e^−7^**	**0.045**	0.607	0.320	0.444				

In bold are highlighted significant values (p *<0.05*). See abbreviations in Tab 2. AIC, Akaike's Information Criterion; AICw, Akaike Information Criterion weight; Rank, model rank from the smallest to the largest AIC value; k, number of variables including the intercept.

Stepwise regression analysis also indicated that presence of guards, research station, tourist station and PA size were the best predictor variables of threat impact level (Table S6).

## Discussion

PAs are fundamental for protecting natural resources and reducing biodiversity loss. It is therefore crucial to document their level of effectiveness against the multitude of threats that they are currently facing [6], [30], [44]. To our knowledge, this study is a first comprehensive attempt to evaluate how specific anthropogenic disturbances influence the ecological viability of PAs in Africa with tropical forest, both at a continental and regional scale, and to analyse which particular conservation actions are best suited to reduce threat impact levels.

### Threats distribution and degree of pressure

Our results indicate that only a few PAs in tropical Africa are better protected, in particular in East Africa, while the rest are under considerable pressure from anthropogenic threats, although the types of threats and degrees of influence vary across regions. According to our analyses, hunting for bushmeat, agriculture and logging are the most common threats with high impact levels.

#### Direct threats

Our findings clearly confirm that wildlife in PAs in tropical Africa is under more pressure from hunting than any other threats [6], [13]. At the continental level, we found no particular differences between the impacts of commercial and subsistence hunting on wildlife populations. Past studies show that hunters who hunt for subsistence needs may often sell their wild game to local markets [45], this was also shown by our correlation study.

However, at a regional level, these hunting activities were found to be most prevalent in West and Central Africa, supporting earlier studies [46], [47]. Commercial hunting in Central Africa occurred at the highest impact level at 36% more in relation to subsistence hunting. In contrast, subsistence hunting in West Africa occurred at the highest level at 15% more than commercial hunting. The combination of these two hunting activities has likely led in the past years to the over-exploitation and even complete disappearance of wildlife in some regions in West and Central Africa, known as the “empty forest syndrome”, where forests are often devoid of large mammals and birds [13], [48], [49]. In fact, over the last decades West and Central Africa have been subject to rapid human population growth and economic development. These along with the improved affordable communications and logistics have increased demand for bushmeat, therefore instigating higher levels of both subsistence and commercial bushmeat [50], [51], [52]. Multiple lines of evidence from West Africa region indicate that hunting has decreased populations of both large and small mammals (e.g., [20],[46],[52]–[54]), fish [44] and large raptors [55]. Instead the majority of PAs in East Africa were under lesser high threat impact levels from hunting. Subsistence hunting was the only type of hunting at the highest level for 7% of PAs. Such a low percentage could be due to a more effective control of hunting or to socio-cultural differences [56].

#### Indirect threats

According to our results, agriculture and logging exerted the highest impact on wildlife in tropical PAs in Africa. In particular at the regional level, the impact of these two land-use types was most prevalent in West Africa, followed by Central and East Africa. These illegal land use activities have long been noted to cause deforestation and forest degradation, to reduce the effective size of PAs and increase faunal decline by facilitating hunters' access to remote areas [6], [57], [58]. Logging often leads to clear-cutting, as it follows an economic value model that is based on initially harvesting the most valuable tree species, and then on extracting less valuable trees [59]. Agriculture will often expands inside PAs because farmers aim to compensate for land that has become unproductive [60].

Settlements within or around a PA were also found to detrimentally impact wildlife at high levels, in particular in West Africa, where they affected almost half of all PAs.

Wildlife in Eastern African PAs were under lesser impact level from indirect threats, with a high prevalence of impacts at levels 1 and 0.

### Conservation activities

Our results generally reinforce a previous study, which found that long-term conservation efforts have a strong positive association with species conservation status [23]. In this study we show that continuous presence of specific conservation activities was associated with the lower threat impact level assessments to PAs.

### Law enforcement

Reduced levels of law enforcement are known to expose wildlife populations within PAs to increased hunting pressure and other illegal activities [23], [61], [62]. The long-term presence of guards was found to have the strongest negative relationship with the threat status to of PAs. Continuous law enforcement patrols were associated with lower impact levels of threats to PAs, but these were of weaker importance compared to other model variables. A patrol usually consists of daily scouts, although evidence shows its effectiveness increases with training, equipment (e.g., guns, patrol vehicles), greater distance covered and with budget [33], [63]. These latter variables were not included in the analyses, because such information was difficult to obtain. The number of guards present was not associated with lower threat impact levels. This may be because in some areas, what matters is not how many guards are present but how effective they are, which is likely to be determined by the strength of their motivation, whether they are well-paid, well-trained and managed, and whether they have the resources to adequately patrol PAs [34], [62], [63], [64]. Lack of data also prevented us from investigating the potential deterring effects of prosecution. Fines and penalties vary across countries in Africa depending on both the level of illegality and on national law. Depending on the seriousness of the crime, violators may be arrested and handed to local authorities. Firearms, ammunition, snares, pit-saw and camping materials may be confiscated or destroyed, along with bushmeat, wood or other resources that have been extracted from the PA [32], [34], [62], [65]. Nevertheless, records for penalising poachers are often poor, because wildlife protection is rarely a national or even local priority [66]. Corruption can often be an additional major problem that tempts poorly paid and resourced park managers, guards and local authorities to disregard law, thus undermining effective conservation programmes [67].

#### Tourism and research

Secondary conservation activities such as tourism and research can have indirect effects for wildlife preservation [68]. We found that high impact threat levels were less prevalent if a PA experienced higher proportions of months when tourism and research stations were active.

Establishing a causal relation is however difficult. On the one hand, both activities may play an important role in deterring hunters, thereby creating “wildlife refugia” [61], [69], [70], [71], in raising employment opportunities and public awareness on the value of conservation, therefore favoring lower threat impact level [72]. On the other hand, one could argue that site security with less pressure from threats may favor the development of both activities, given for instance higher presence of wildlife that make their viewing easier and attractive to both tourists and researchers.

The size of a PA had positive relationship on threat impact levels. Past studies focussing on this variable have demonstrate that PAs with smaller size are more vulnerable to threats and experience an accelerated habitat loss and wildlife extinction rates in contrast to large areas [22], [73].

## Conclusion and recommendations

Overall, our study highlights deficiencies in the effectiveness of conservation activities in controlling threats to the wildlife of several PAs across tropical Africa. Our findings support existing evidence that the majority of African PAs are in a critical state. At a regional level, East African PAs are under less threats pressure. In contrast, Central and West African PAs are under significant anthropogenic pressure, in particular Western PAs where several threatened wildlife species range in endemic habitat and the investment in conservation management is extremely low [74], [18]. Among the 12 threats considered, hunting (for commercial and subsistence purposes) and habitat degradation (agriculture and logging) were the most intense.

Large mammals and birds are usually among the first animals to be affected by these threats [52], [64], [75]. An over-exploitation and destruction of their habitat is likely to lead them to local and global extinction. Many of these animals are key for the survival of the ecosystem they inhabit, given their role in seed dispersal [32], [76], [77]. Their extinction in tropical forest is likely to cause significant cascade effects across the trophic web, thus causing secondary or co-extinctions and consequently severe and irreversible changes to the ecosystem functioning [78].

Moreover, this study provides a first comprehensive continent-wide evaluation of the relative importance of conservation activities against in mitigating threat impact level for the protection of PAs in tropical Africa. Our findings show how threat impact level on wildlife in a PA in tropical Africa is negatively influenced by the continuous presence of conservation activities, such as guards, tourism and research stations. This leads us to suggest the following priorities for management decisions: (i) conservation activities should be sustained over the long-term and that (ii) more sites for research and tourism could be developed as additional benefits for the protection of wildlife. Such measures are only possible with the provision of substantial long-term financial support and the full involvement of local, national, and international stakeholders. Moreover, conservation efforts will benefit from the training of national and international students and researchers, as well as the involvement of local communities.

Although our study has not been able to measure an absolute level of impact on the ground, it is a first quantified assessment of threat levels. An evidence-based approach can allow quantifying and monitoring the success of such conservation activities. PAs are one of the key conservation elements to conserve wildlife, habitats, landscapes, and benefit local communities. Continuous and rigorous monitoring on the ground is likely to increase the effectiveness of protection activities over time by aiding adaptive management to reduce threats, to assess wildlife status, and to improve park management.

## Supporting Information

Figure S1 **Threats and their impact level (1, 2, 3) in the different countries and the number of PAs where they occur.**
(TIF)

Figure S2 **Dendrogram showing the grouping of threats.**
(TIF)

Table S1 **List of the 98 PAs.** PA_ID is the protected area identity code. The current name of the PAs is not provided due to confidentiality agreements with some data providers. The code is composed by a two-letter ISO (International Organization for Standardization) code and by a number (lowest to the highest value according to their regional location from west to east). The PA size is expressed per km^2^.
(DOC)

Table S2 **Questionnaire template on threats impact level affecting biodiversity within protected areas.**
(DOC)

Table S3 **Questionnaire template on conservation activities.**
(DOC)

Table S4 **Field description section on questionnaires related to conservation activities.**
(DOC)

Table S5 **Pearson correlations between all conservation activities.** (in bold are correlations with rho>0.50 and p <0.0001)
(DOC)

Table S6 **Stepwise regression results.**
(DOC)

Text S1 **Additional information on the methods section.**
(DOC)
