# Supplementary figures and images for: Protected Areas in Tropical Africa: Assessing Threats and Conservation Activities
Source: PLoS One. 2014 Dec 3;9(12):e114154. doi: 10.1371/journal.pone.0114154 (PMC4254933; doi:10.1371/journal.pone.0114154)

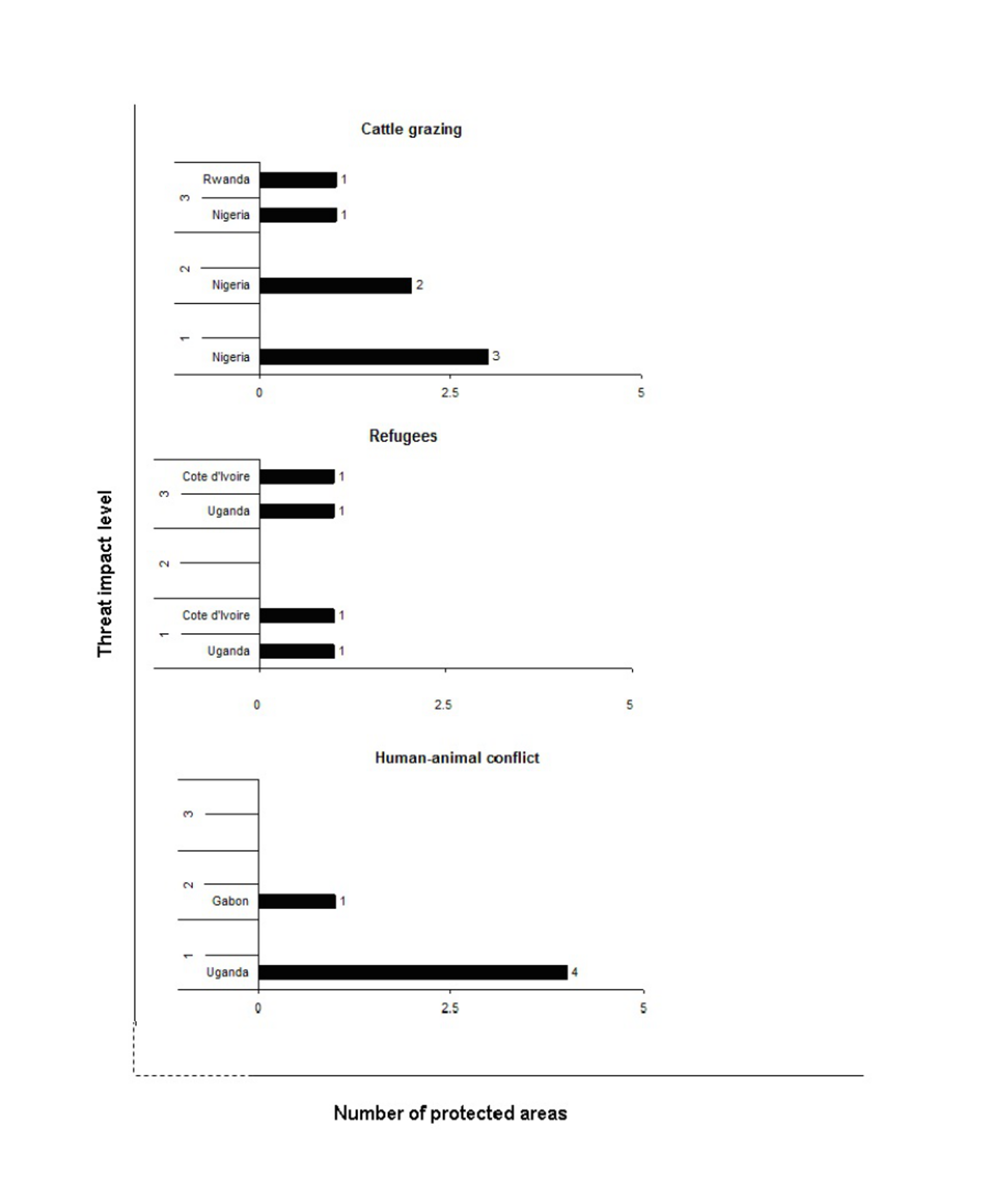

Supplement: Figure S1 — Threats and their impact level (1, 2, 3) in the different countries and the number of PAs where they occur. (TIF) [file pone.0114154.s001.tif]

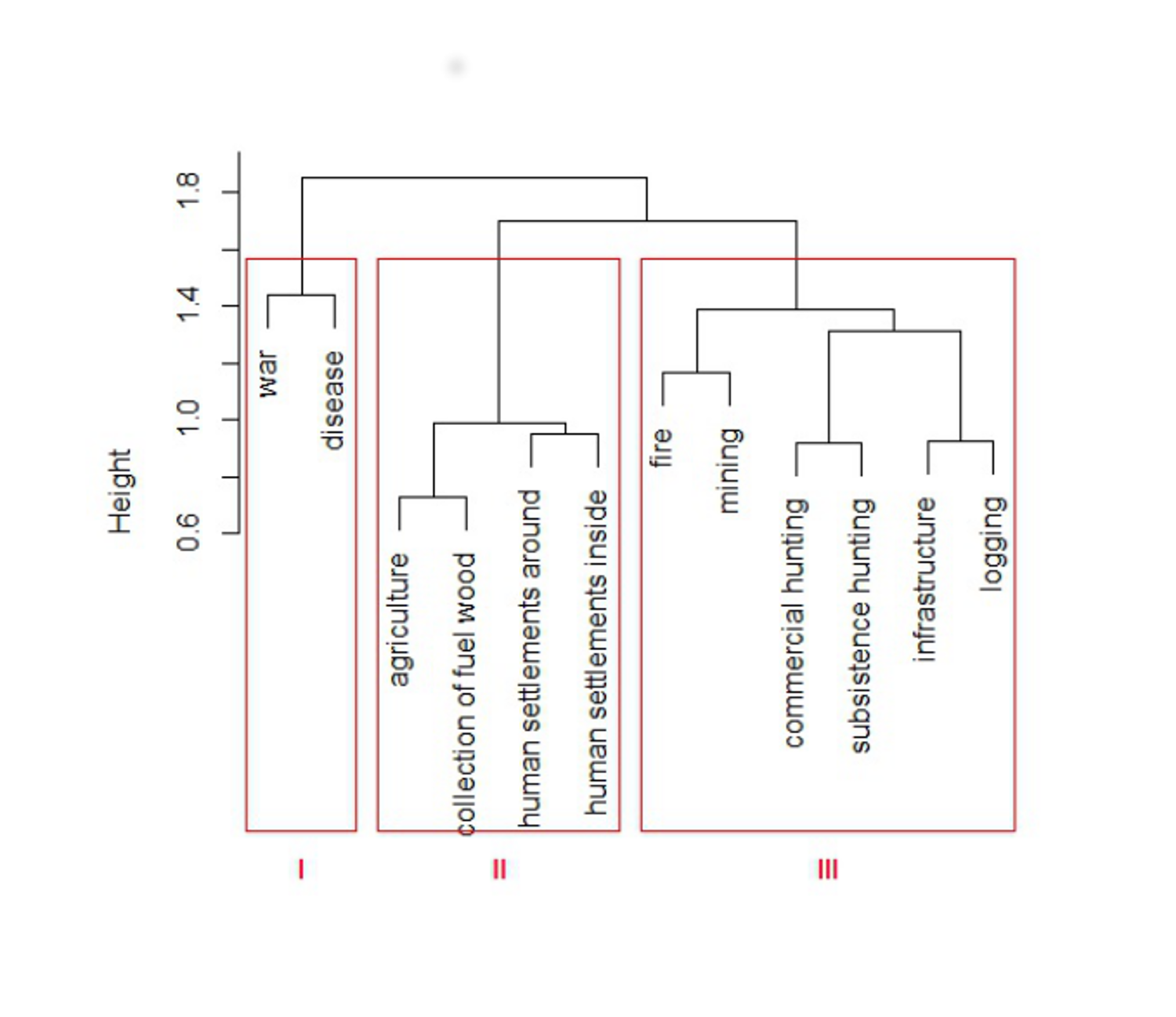

Supplement: Figure S2 — Dendrogram showing the grouping of threats. (TIF) [file pone.0114154.s002.tif]
