# Supplementary material for: Protected Areas in Tropical Africa: Assessing Threats and Conservation Activities
Source: PLoS One. 2014 Dec 3;9(12):e114154. doi: 10.1371/journal.pone.0114154 (PMC4254933; doi:10.1371/journal.pone.0114154)
Supplement: Table S1 — List of the 98 PAs. PA_ID is the protected area identity code. The current name of the PAs is not provided due to confidentiality agreements with some data providers. The code is composed by a two-letter ISO (International Organization for Standardization) code and by a number (lowest to the highest value according to their regional location from west to east). The PA size is expressed per km2. (DOC) [file pone.0114154.s003.doc]

| **Region** | **Country** | **PA_ID** | **Size** |
| --- | --- | --- | --- |
| Western | Guinea | GN_01 | 278.19 |
| Western | Guinea | GN_02 | 598.46 |
| Western | Guinea | GN_03 | 791.62 |
| Western | Guinea | GN_04 | 147.42 |
| Western | Guinea | GN_05 | 572.00 |
| Western | Guinea | GN_06 | 616.62 |
| Western | Guinea | GN_07 | 922.92 |
| Western | Sierra Leone | SL_08 | 90.00 |
| Western | Sierra Leone | SL_09 | 141.00 |
| Western | Sierra Leone | SL_10 | 840.61 |
| Western | Sierra Leone | SL_11 | 397.57 |
| Western | Sierra Leone | SL_12 | 129.00 |
| Western | Sierra Leone | SL_13 | 841.00 |
| Western | Sierra Leone | SL_14 | 116.00 |
| Western | Sierra Leone | SL_15 | 454.00 |
| Western | Sierra Leone | SL_16 | 151.63 |
| Western | Côte d’Ivoire | CI_17 | 197.82 |
| Western | Côte d’Ivoire | CI_18 | 782.68 |
| Western | Côte d’Ivoire | CI_19 | 1349.30 |
| Western | Côte d’Ivoire | CI_20 | 62.57 |
| Western | Côte d’Ivoire | CI_21 | 313.42 |
| Western | Côte d’Ivoire | CI_22 | 916.09 |
| Western | Côte d’Ivoire | CI_23 | 228.48 |
| Western | Côte d’Ivoire | CI_24 | 11963.64 |
| Western | Côte d’Ivoire | CI_25 | 112.65 |
| Western | Côte d’Ivoire | CI_26 | 630.84 |
| Western | Côte d’Ivoire | CI_27 | 511.76 |
| Western | Côte d’Ivoire | CI_28 | 1035.67 |
| Western | Côte d’Ivoire | CI_29 | 32829.80 |
| Western | Côte d’Ivoire | CI_30 | 214.91 |
| Western | Côte d’Ivoire | CI_31 | 230.16 |
| Western | Côte d’Ivoire | CI_32 | 939.05 |
| Western | Côte d’Ivoire | CI_33 | 113.44 |
| Western | Côte d’Ivoire | CI_34 | 5385.57 |
| Western | Côte d’Ivoire | CI_35 | 284.64 |
| Western | Ghana | GH_36 | 511.74 |
| Western | Ghana | GH_37 | 308.54 |
| Western | Ghana | GH_38 | 121.72 |
| Western | Ghana | GH_39 | 45.43 |
| Western | Ghana | GH_40 | 175.00 |
| Western | Ghana | GH_41 | 482.28 |
| Western | Ghana | GH_42 | 48.12 |
| Western | Ghana | GH_43 | 190.12 |
| Western | Ghana | GH_44 | 211.22 |
| Western | Nigeria | NG_45 | 103.87 |
| Western | Nigeria | NG_46 | 309.93 |
| Western | Nigeria | NG_47 | 239.98 |
| Western | Nigeria | NG_48 | 393.65 |
| Western | Nigeria | NG_49 | 670.29 |
| Western | Nigeria | NG_50 | 335.50 |
| Western | Nigeria | NG_51 | 5876.43 |
| Western | Nigeria | NG_52 | 559.90 |
| Western | Nigeria | NG_53 | 270.96 |
| Western | Nigeria | NG_54 | 45.50 |
| Western | Nigeria | NG_55 | 85.98 |
| Western | Nigeria | NG_56 | 144.24 |
| Western | Nigeria | NG_57 | 827.62 |
| Western | Nigeria | NG_58 | 1326.68 |
| Western | Nigeria | NG_59 | 309.70 |
| Central | Cameroon | CM_60 | 17.64 |
| Central | Cameroon | CM_61 | 189.91 |
| Central | Cameroon | CM_62 | 9.30 |
| Central | Cameroon | CM_63 | 3336.23 |
| Central | Cameroon | CM_64 | 2625.55 |
| Central | Cameroon | CM_65 | 5878.37 |
| Central | Cameroon | CM_66 | 850.11 |
| Central | Cameroon | CM_67 | 1308.31 |
| Central | Cameroon | CM_68 | 2173.50 |
| Central | Cameroon | CM_69 | 456.42 |
| Central | Central African Republic | CF_70 | 2140.35 |
| Central | Central African Republic | CF_71 | 1251.02 |
| Central | Equatorial Guinea | GQ_72 | 1938.50 |
| Central | Gabon | GA_73 | 1495.28 |
| Central | Gabon | GA_74 | 4948.12 |
| Central | Gabon | GA_75 | 1201.65 |
| Central | Gabon | GA_76 | 967.62 |
| Central | Gabon | GA_77 | 1068.44 |
| Central | Congo | CG_78 | 4529.85 |
| Central | Congo | CG_79 | 4074.18 |
| Central | Democratic Republic of Congo | CD_80 | 32967.23 |
| Central | Democratic Republic of Congo | CD_81 | 6134.99 |
| Central | Democratic Republic of Congo | CD_82 | 6720.87 |
| Central | Democratic Republic of Congo | CD_83 | 481.52 |
| Central | Democratic Republic of Congo | CD_84 | 11105.42 |
| Eastern | Uganda | UG_85 | 816.27 |
| Eastern | Uganda | UG_86 | 399.52 |
| Eastern | Uganda | UG_87 | 86.81 |
| Eastern | Uganda | UG_88 | 185.44 |
| Eastern | Uganda | UG_89 | 139.85 |
| Eastern | Uganda | UG_90 | 383.94 |
| Eastern | Uganda | UG_91 | 789.71 |
| Eastern | Uganda | UG_92 | 445.15 |
| Eastern | Uganda | UG_93 | 996.45 |
| Eastern | Uganda | UG_94 | 221.13 |
| Eastern | Rwanda | RW_96 | 271.21 |
| Eastern | Rwanda | RW_97 | 1017.24 |
| Eastern | Burundi | BI_98 | 15.27 |
| Eastern | Burundi | BI_99 | 373.93 |
