# Supplementary material for: Protected Areas in Tropical Africa: Assessing Threats and Conservation Activities
Source: PLoS One. 2014 Dec 3;9(12):e114154. doi: 10.1371/journal.pone.0114154 (PMC4254933; doi:10.1371/journal.pone.0114154)
Supplement: Table S2 — Questionnaire template on threats impact level affecting biodiversity within protected areas. (DOC) [file pone.0114154.s004.doc]

| **Site Name:** | year | year | year | year | year |
| --- | --- | --- | --- | --- | --- |
| Threats rank (0=threat absent; 1=low impact, threat present with minimal impact on wildlife populations; 2=moderate impact, threat present and affecting wildlife populations but impact not critical to their survival; 3= high impact, threat present and critically affecting survival of wildlife populations) |  |  |  |  |  |
| Commercial hunting |  |  |  |  |  |
| Subsistence Hunting |  |  |  |  |  |
| Agriculture |  |  |  |  |  |
| Fuel wood |  |  |  |  |  |
| Infrastructure (roads, etc) |  |  |  |  |  |
| Human settlements around |  |  |  |  |  |
| Human settlements inside |  |  |  |  |  |
| War |  |  |  |  |  |
| Disease |  |  |  |  |  |
| Fire |  |  |  |  |  |
| Mining |  |  |  |  |  |
| Logging |  |  |  |  |  |
| other threats (please list) |  |  |  |  |  |
