# Supplementary material for: Protected Areas in Tropical Africa: Assessing Threats and Conservation Activities
Source: PLoS One. 2014 Dec 3;9(12):e114154. doi: 10.1371/journal.pone.0114154 (PMC4254933; doi:10.1371/journal.pone.0114154)
Supplement: Table S3 — Questionnaire template on conservation activities. (DOC) [file pone.0114154.s005.doc]

| **Site Name:** | year | year | year | year | year |
| --- | --- | --- | --- | --- | --- |
|  |  |  |  |  |  |
| Yes/No law enforcement guards |  |  |  |  |  |
| Number law enforcement guards |  |  |  |  |  |
| Yes/No guard patrols per month |  |  |  |  |  |
| Number guard patrols per month |  |  |  |  |  |
| Comments |  |  |  |  |  |
|  |  |  |  |  |  |
| Yes/No Tourism |  |  |  |  |  |
| Yes/No active tourism station (specify months of duration) |  |  |  |  |  |
| Number tourists per month |  |  |  |  |  |
| Number tourists per year |  |  |  |  |  |
| Comments |  |  |  |  |  |
|  |  |  |  |  |  |
| Yes/No research program |  |  |  |  |  |
| Yes/No active research station (specify months of duration) |  |  |  |  |  |
| Comments |  |  |  |  |  |
