# Supplementary material for: Protected Areas in Tropical Africa: Assessing Threats and Conservation Activities
Source: PLoS One. 2014 Dec 3;9(12):e114154. doi: 10.1371/journal.pone.0114154 (PMC4254933; doi:10.1371/journal.pone.0114154)
Supplement: Table S4 — Field description section on questionnaires related to conservation activities. (DOC) [file pone.0114154.s006.doc]

| **Question** | **Definition** |
| --- | --- |
|  |  |
| Yes/No law enforcement guards | Presence or absence of law enforcement guards employed for the protection of the area |
| Number law enforcement guards | Average number of law enforcement guards working for the protection of the area |
| Yes/No guard patrols per month | Presence or Absence of guards patrolling the area during the period of a month |
| Number guard patrols per month | Average number of patrols made by law enforcement guards during the period of a month |
| Comments |  |
|  |  |
| Yes/No Tourism | Presence/absence of tourism in the area |
| Yes/No active tourism station (specify months of duration) | Presence/absence of tourist station used for present tourists (average number of month of duration) |
| Number tourists per month | Average number of tourists per month |
| Number tourists per year | Average number of tourists per year |
| Comments |  |
|  |  |
| Yes/No research program (specify months of duration) | Presence/absence of research study conducted in the area (average number of month of duration of the study) |
| Yes/No active research station (specify months of duration) | Presence/absence of research station used by researchers (average number of month of duration) |
| comments |  |
