# Supplementary material for: Protected Areas in Tropical Africa: Assessing Threats and Conservation Activities
Source: PLoS One. 2014 Dec 3;9(12):e114154. doi: 10.1371/journal.pone.0114154 (PMC4254933; doi:10.1371/journal.pone.0114154)
Supplement: Table S5 — Pearson correlations between all conservation activities. (in bold are correlations with rho>0.50 and p <0.0001) (DOC) [file pone.0114154.s007.doc]

|  | **Guards** | **Number of guards** | **Guards monthly patrol** | **Research site** | **Research station** | **Tourism site** | **Tourist station** | **Number of tourists** |
| --- | --- | --- | --- | --- | --- | --- | --- | --- |
| **Guards** | 1.00 |  |  |  |  |  |  |  |
| **Number of guards** | **0.94** | 1.00 |  |  |  |  |  |  |
| **Guards monthly patrol** | **0.84** | **0.87** | 1.00 |  |  |  |  |  |
| **Research site** | 0.41 | 0.39 | 0.37 | 1.00 |  |  |  |  |
| **Research station** | 0.16 | 0.14 | 0.21 | **0.63** | 1.00 |  |  |  |
| **Tourism site** | **0.53** | **0.56** | **0.66** | 0.37 | 0.18 | 1.00 |  |  |
| **Tourist station** | **0.53** | **0.57** | **0.64** | **0.63** | 0.19 | 0.18 | 1.00 |  |
| **Number of tourists** | **0.52** | **0.55** | **0.64** | 0.31 | 0.19 | **0.98** | **0.88** | 1.00 |
