# Supplementary material for: Protected Areas in Tropical Africa: Assessing Threats and Conservation Activities
Source: PLoS One. 2014 Dec 3;9(12):e114154. doi: 10.1371/journal.pone.0114154 (PMC4254933; doi:10.1371/journal.pone.0114154)
Supplement: Table S6 — Stepwise regression results. (DOC) [file pone.0114154.s008.doc]

|  | | **Parameters** | | | | | | |
| --- | --- | --- | --- | --- | --- | --- | --- | --- |
| **Full models** | **Sub-models** | **Sum of Sq** | **RSS** | **Sub-modelAIC** | **Full model AIC** | **AICw** | **Rank** | **k** |
| G+T+RS+TS+S* | G+RS+TS+S | 0.029 | 7.207 | -169.03 | -167.34 | 0.077 | 1 | 5 |
|  | Intercept |  | 7.178 | -167.34 | -167.34 | 0.033 | 2 | 1 |
|  | T+RS+TS+S | 0.241 | 7.419 | -166.83 | -167.34 | 0.025 | 3 | 5 |
|  | G+T+RS+TS | 0.291 | 7.469 | -166.32 | -167.34 | 0.020 | 4 | 5 |
|  | G+T+TS+S | 0.660 | 7.838 | -162.65 | -167.34 | 0.003 | 5 | 5 |
|  |  |  |  |  |  |  |  |  |
| G+RS+TS+S**^,§^ | Intercept |  | 7.207 | -169.03 | -169.03 | 0.099 | 1 | 1 |
|  | G+RS+TS | 0.286 | 7.493 | -168.07 | -169.03 | 0.061 | 2 | 4 |
|  | RS+ TS +S | 0.311 | 7.519 | -167.81 | -169.03 | 0.054 | 3 | 4 |
|  | G+RS+S | 0.335 | 7.542 | -167.58 | -169.03 | 0.048 | 4 | 4 |
|  | G+RS+TS+S+T | 0.030 | 7.178 | -167.34 | -169.03 | 0.042 | 5 | 6 |
|  | G+TS+S | 0.665 | 7.873 | -164.32 | -169.03 | 0.009 | 6 | 4 |
