# Supplementary material for: Protected Areas in Tropical Africa: Assessing Threats and Conservation Activities
Source: PLoS One. 2014 Dec 3;9(12):e114154. doi: 10.1371/journal.pone.0114154 (PMC4254933; doi:10.1371/journal.pone.0114154)
Supplement: Text S1 — Additional information on the methods section. (DOC) [file pone.0114154.s009.doc]

**Supplementary Text**

**Literature Research**

A search of publications on local threats to wildlife and conservation activities in protected areas (PAs) was performed using the following sources: web search engines (such as the "ISI web of science" and "Google scholar"). In order to optimize the search process through web search engines, a standard protocol was followed based on the use of specific keywords in the search engines (see Appendix A1 for details). These keywords would then appear both in the title and body text of the paper. Papers with relevant data were then selected.

Information on conservation activities included a total of 2,041 references. These sources cover the year and type of conservation activity at each PA (i.e. law enforcement guard, tourism, research). The quality of data on conservation efforts was assumed to be accurate as it was based upon scientific field-research. The extracted data and its source were standardized and recorded on pre-designed spreadsheets.

**Questionnaires**

Employing questionnaires to gather qualitative data is widely used in assessing conservation effectiveness [1,2]. Thus a questionnaire was designed and employed to gather information about local threats to wildlife of 256 PAs (Supplementary Table S2). This was presented to field researchers and park managers with long-term field and empirical data collection experience (working in the field for more than one year), through a variety of mediums, including telephone and email, as well as face-to-face meetings. It was presented to 192 possible respondents. The overall response rate was 75%, including fully or partially completed questionnaires. Questions varied according to when and where respondents worked / currently work, and their role in conservation (e.g., working for wildlife surveys, park management).

The structure of the questionnaire was designed to make it as simple as possible for the interviewee. The question was clear and brief, requiring a numerical response. To ensure that the aims and meanings of each question were clear, questionnaires included a detailed "field description" section in a separate excel sheet (Table 1 in main text). Questionnaires were sent to more than two individuals working in a given area during the same period. On the occasions where interviewees provided conflicting responses, the most frequent answer (>50% of the total) was included in the analysis. The same procedure was followed for questionnaires related to conservation activities sent to long-term field experts and researchers to fill gaps in information for specific PAs (Supplementary Table S3 and Supplementary Table S4 for field description reported in a separate excel sheet). In order to test the consistency of responses we ran a Coefficient Alpha Reliability Test for each PA [3]. The results show an Alpha value that ranged between 0.70 to 0.99, demonstrating a good internal consistency.

Our main concern about the responses to the questionnaires is that they may be biased due to professional or political reasons. For this reason, we asked the respondents if they wished to not be included as co-authors in the manuscript or not to publish the name of the PA. The majority of respondents did not have concerns about being a co-author, however some requested a confidentiality agreement that states to not report or mention the names and specific locations of their PAs because of possible political or professional retributions. Before the use of their data for the analysis of this paper, we agreed that if an outside researcher wishes to know the name for a particular area or more, he/she can contact directly the first co-author and leader of this study (Dr Sandra Tranquilli: [sandra.tranq@gmail.com](mailto:sandra.tranq@gmail.com)) who will provide the contact details of the data provider for the specific PA.

**Appendix 1**

**Keywords used in the web search engines**

- Information on local threats was performed using the following keywords: threats to wildlife, name of PA, name of country, name of region and PA status (e.g., national park, natural reserve).
- Information on conservation activities was performed using the following keywords used as single words: law enforcement guards, law enforcement patrol, law enforcement number, tourism, tourists, tourist station, research, research station, researchers. These were also combined with the name of PA, name of country and PA status (e.g., national park, natural reserve).

**Supplemental References**

1. Ervin J (2003) Rapid assessment of protected area management effectiveness in four countries. BioSci 53: 833-841.
2. Struhsaker TT, Struhsaker PJ, Siex KS (2005) Conserving Africa's rain forests: problems in protected areas and possible solutions. Biol Conserv 123: 45-54.
3. Cronbach LJ (1951) Coefficient alpha and the internal structure of tests. Psychometrika 16: 297-334.
